# Solid waste motor tricycle operators in Kumasi, Ghana, harbour respiratory pathogens; a public health threat

**DOI:** 10.1371/journal.pone.0284985

**Published:** 2023-04-24

**Authors:** Stephen Yaw Armoh, Sherihane Aryeetey, Japhet Senyo Kamasah, Kennedy Gyau Boahen, Michael Owusu, Augustina Adjei-Boateng, Olivia Agbenyega, Alexander Kwarteng, Suzanne Hingley-Wilson, Kwasi Obiri-Danso, Daniel Ansong, Augustina Angelina Sylverken

**Affiliations:** 1 Department of Theoretical and Applied Biology, Kwame Nkrumah University of Science and Technology, Kumasi, Ghana; 2 Kumasi Centre for Collaborative Research in Tropical Medicine, Kwame Nkrumah University of Science and Technology, Kumasi, Ghana; 3 Department of Molecular Medicine, Kwame Nkrumah University of Science and Technology, Kumasi, Ghana; 4 Department of Clinical Microbiology, Kwame Nkrumah University of Science and Technology, Kumasi, Ghana; 5 Department of Medical Diagnostics, Kwame Nkrumah University of Science and Technology, Kumasi, Ghana; 6 Research and Development Unit, Waste Management Department, Kumasi Metropolitan Assembly, Kumasi, Ghana; 7 Department of Agroforestry, Kwame Nkrumah University of Science and Technology, Kumasi, Ghana; 8 Department of Biochemistry and Biotechnology, Kwame Nkrumah University of Science and Technology, Kumasi, Ghana; 9 Department of Microbial Sciences, Faculty of Health and Medical Science, University of Surrey, Guildford, United Kingdom; 10 Department of Child Health, Kwame Nkrumah University of Science and Technology, Kumasi, Ghana; Satyawati College, University of Delhi, INDIA

## Abstract

**Background:**

The use of motor tricycles in transporting municipal solid waste (MSW) within urban and peri-urban towns in Ghana is on the increase. This activity often leads to the introduction of pathogen-containing bioaerosols into the environment, as well as to the tricycle operators. We sought to investigate the prevalence and associated risk factors of respiratory pathogens among solid waste tricycle operators.

**Methods:**

A cross-sectional study was conducted among 155 solid waste transporters who use motor tricycles using semi-structured interviews. Nasopharyngeal swabs were obtained from participants and screened for respiratory pathogens using Polymerase Chain Reaction (PCR).

**Results:**

Pathogens detected in participants were SARS-CoV-2 (n = 10, 6.5%) and *Streptococcus pneumoniae* (n = 10, 6.5%), constituting an overall prevalence of 12.9% and co-infection rate of 1.3%. The most common self-reported symptoms were cough (n = 67, 43.2%), sore throat (n = 44, 28.4%) and difficulty in breathing (n = 22, 14.2%). Adherence to the use of gloves (n = 117, 75.5%) and nose mask (n = 110, 71.0%) was high. There was a significant association between the detection of respiratory pathogens and the use of gloves, use of more than one PPE and exposure to other pollutants (p < 0.05). Individuals who were exposed to “other pollutants” significantly had lower odds of becoming infected with respiratory pathogens (Adj. OR (95% CI): 0.119(0.015,0.938).

**Conclusion:**

Although prevalence of respiratory pathogens is generally low, strict adherence to PPE use could further reduce its rates to even lower levels. Governmental health institutions and informal solid waste transporters should address challenges related to exposure to pollutants, use of gloves, and multiple PPE.

## Introduction

In recent times, the use of motor tricycles as a means of transport has been on the rise [1]. In many Low to Middle-income countries (LMIC), including Ghana, motor tricycles have become a convenient means for transporting individuals, farm produce, general goods and even carting solid waste [2]. The use of motor tricycles to transport solid waste has become highly patronized within major cities in Ghana as the cost of these tricycles is relatively cheaper compared to garbage trucks owned by authorized companies [3]. Also, these motor tricycles can transport solid waste from densely populated communities with poor road networks which cannot be easily accessed by heavy-duty garbage trucks [4].

The use of motor tricycles as a means of transporting solid waste poses a public health issue. These tricycles are not designed with a protective compartment to conceal the refuse as they are being transported to landfill sites. Thus, bioaerosols which may consist of bacteria, viruses and other harmful microorganisms may be released from the refuse into the environment. In addition, most solid waste tricycle operators do not put on the appropriate personal protective equipment (PPE) while transporting carting the solid waste [4, 5]. The work of solid waste tricycle operators is worrisome as their health could suffer, they could serve as reservoirs of respiratory pathogens and conduits in spreading these pathogens as refuse is transported openly within the communities. Such activities may lead to the transmission of respiratory pathogens such as *Haemophilus influenzae*, *Neisseria meningitides*, *Streptococcus pneumoniae*, *Influenza viruses* among others [6].

Asibey *et al*., [5] assessed the occupational health and safety among solid waste motor tricycle operators in Kumasi. In this study, they found very low adherence to safety precautions and awareness of risks and observed risky practices of collecting waste with bare hands and not using nose masks and coveralls, practices which predisposes them to respiratory infections [7]. However, little is known about the proportion of motor tricycle operators who harbour common respiratory pathogens. This study, therefore, aimed to determine the proportion of solid waste motor tricycle operators within the Kumasi Metropolis habouring respiratory pathogens and further investigate some of the risk factors that influence exposure to these pathogens. Such information could provide the basis to advocate for legislation and interventions on how solid waste transporters in Ghana and other parts of the world can protect themselves and others from being infected with microbes in their line of work.

## Materials and methods

### Study design and setting

This was a cross-sectional study aimed at estimating the burden of selected respiratory pathogens among selected operators of motor tricycles who transport refuse in the Kumasi metropolis (KMA). It was also important to determine some of the risk factors that predispose them to these pathogens. KMA is one of 43 Metropolitan, Municipal, and District Assemblies (MMDAs) in the Ashanti Region of Ghana. This area was chosen purposively because the KMA has challenges and inefficiencies in solid waste management [3], which has led to the emergence of informal motor tricycle solid waste collectors. The operations of the informal motor tricycle solid waste collectors supplement attempts by city authorities’ to manage solid waste particularly in low-income informal communities [5].

### Ethical approval

The study was approved by the Committee on Human Research Publications and Ethics (CHRPE) of the School of Medicine and Dentistry of the Kwame Nkrumah University of Science and Technology (KNUST) and the Komfo Anokye Teaching Hospital (KATH), Kumasi (CHPRE No. CHRPE/AP/333/20). Permission was also sought from the Kumasi Metropolitan Assembly. Written informed consent was obtained from each subject after the study protocol was explained to them.

### Sampling and sample size

The selection of study participants was done based on convenience. A sample size of 147 was estimated based on an assumed prevalence of 36% of respiratory pathogens [8], an estimated 250 informal tricycle solid waste collectors, a 5.0% margin of error, and a 95% confidence level.

### Data collection

Before the start of the study, focus group discussions were organized with the leaders of the solid waste motor tricycle operators. Five participants were purposively selected based on their level of experience (number of years they had operated as motor tricycle operators) for the discussion. The discussion focused on general health problems, concerns about compliance with safety precautions, and some general concerns about their work, health and safety. Questionnaires were pre-tested before implementation in the field. The questionnaire was revised based on the suggestions and comments from the pre-testing. Structured questionnaires were administered to study participants as data collecting instruments. The questionnaires captured the basic demographic characteristics, frequency of transporting, symptoms experienced and smoking history among other variables. The questionnaire administration was done using the CommCare cloud computing software package (Dimagi Inc., USA).

### Sample collection

Two nasopharyngeal specimens were taken from each subject using flocked swabs (BioTeke Corporation, China). The sample was obtained by gently inserting the swab up the nostril towards the throat until resistance was felt, and then rotating the swab three times to acquire epithelial cells. The swabs intended for viral detection were immediately transported to the laboratory in 1.5 mL tubes containing viral transport medium (BioTeke Corporation, China). All samples were transported to the laboratory in a cold chain (2 to 8°C) and stored at– 70°C until testing.

### Nucleic acid extraction PCR testing

Nucleic acid was extracted from the samples using the Guangzhou LBP Medicine Combo extraction kit (Guangzhou LBP Medicine, China) and following the manufacturer’s instructions. Polymerase Chain Reaction (PCR) was performed according to the method described in [9], with some modifications in the total reaction volume, the volume of tenfold-concentrated polymerase synthesis buffer (10X buffer), the volume of nuclease-free water and the introduction of an initial denaturation stage in the cycling conditions. PCR was performed in a 25 μL reaction volume, containing 0.4 μM of primers (biopolymer company) (both forward and reverse) autolysin gene *lytA* from *Streptococcus pneumoniae* (Table 1), 150 μM of each deoxyribonuclease triphosphate (dNTPs) (Invitrogen, USA), 1.5 mM of MgCl_2_ (Qiagen, Germany)_,_ 2.5 μL of tenfold-concentrated polymerase synthesis buffer (10X buffer), 1.5 U of Taq DNA polymerase (BOLIS, BIODYME), 2 μL of DNA template and 12.875 μL of nuclease-free water. To perform the amplification, 23 μL of the master mixture was added to PCR tubes after which 2 μL of DNA template was added. The PCR tubes containing the master mixture and DNA template were placed on Thermocycler (ThermoFisher Scientific, USA) which had been programmed according to the cycling protocol. The PCR conditions were as follows: initial denaturation at 95°C for 15 s, denaturation for 30 s at 95°C, annealing for 30 s at 60°C, and extension for 60 s at 72°C for 40 cycles. The PCR products were separated on a 0.5% (wt/vol) agarose gel (Sigma, USA) for 25 min at 80 voltage and visualized by illumination with UV light after ethidium bromide staining. A 100-bp DNA ladder (Invitrogen, USA) was used as an external fragment size standard.

**Table 1:** Target genes and primer sequences for respiratory pathogens.

Organism (Target gene/region)	Function	Oligonucleotide sequence	References
***Streptococcus pneumoniae*** **(*lytA* (395bp))**	Forward Reverse	GGCTACTGGTACGTACATTC AATCAAGCCATCTGGCTCTA	[9]
**SARS-CoV-2** **(ORF1ab (RdRp) gene** **& E gene)**	nCoV_2019 Forward nCoV_2019 Reverse nCoV_2019 Probe E_Sarbeco_Forward E_Sarbeco_Reverse E_Sarbeco_Probe	CAAATTCTATGGTGGTTGGCACA GGCATGGCTCTATCACATTTAGG FAM- ATAATCCCAACCCATRAG-MGB ACAGGTACGTTAATAGTTAATAGCGT ATATTGCAGCAGTACGCACACA FAM- ACACTAGCCATCCTTACTGCGCTTCG-BHQ1	[11, 12]
**Influenza A (H_3_N_2_)** **(Hemagglutinin)**	Flu A universal forward primer Flu A universal reverse primer Flu A probe H3 forward primer l H3 reverse primer H3 probe	5’-GACCRATCCTGTCACCTCTGAC-3’ 5’-GGGCATTYTGGACAAAKCGTCTACG-3’ 5’-HEX-TGCAGTCCTCGCTCACTGGGCACG-BHQ1-3’ 5’-ACCCTCAGTGTGATGGCTTCCAAA-3’ 5’-TAAGGGAGGCATAATCCGGCACAT-3’ 5’-CY5-ACGCAGCAAAGCCTACAGCAACTGT-BHQ3-3’	[13]

Real-time reverse transcription-polymerase chain reaction (RT-PCR) of SARS-CoV-2 RNA detection was performed using Standard M nCoV real-time detection kit (SD Biosensor Inc., Republic of Korea) which targets the ORF1ab (RdRp) gene and E gene in SARS-CoV-2 (**Table 1**) following the manufacturer’s instructions. Samples were screened for Influenza A (H_3_N_2_) using the method described by Van Elden *et al*., [10].

### Data management and analysis

Data were double-entered, aggregated, and cleaned in Microsoft Excel (version 2019). For statistical analysis, the Statistical Package for the Social Sciences (IBM-SPSS) (version 23) was used. Descriptive analysis was used to determine the frequency and percentages of categorical variables. Fisher’s exact test or, when appropriate, the Chi-square test was used to determine the relationship between respiratory pathogen detection and independent variables. Significant variables in the Chi-square test were entered into a logistic regression model. Estimates were given as an Odds Ratio (OR) and a 95% confidence interval. Multivariate logistic regression analysis was used to determine the independent effects of associated factors on respiratory infection. A result was considered statistically significant when the p-value was ≤ 0.05.

## Results

### Characteristics of waste motor tricycle operators

A total of 155 participants were enrolled in this study and were all males. The mean age of the study population was 25.8 ± 7.6 years (16 – 60years). Of the participants, 133 (85.5%) had little or no formal education (< SHS/Tech/Voc.), whiles 22 (14.2%) had a higher level of education. A total of 117 (75.5%) of the respondents had worked as motor tricycle operators for 5 or fewer years. Most of the participants (141; 91.0%) transported solid waste to the landfill sites once daily. About a third of the motor tricycle operators were asymptomatic (49; 31.6%). Of the 106 subjects who were symptomatic, they presented with symptoms like cough 67 (43.2%), sore throat 44 (28.4%), difficulty in breathing 22 (14.2%), chronic mucus 14 (9%), chest pain 12 (7.7%), persistent cough 5 (3.2%) and coughing up blood had 1 (0.6%) (**Table 2**).

**Table 2:** Socio-demographic and clinical characteristics of subjects.

	Negative	Positive	Total	*p-value*
Total	137	18	155	
**Age of subjects**				0.132
< 24	58 (42.3)	11 (61.1)	69 (44.5)	
≥ 24	79 (57.6)	7 (38.9)	86 (55.5)	
**Work experience (years)**				0.410
≤ 5	102 (74.5)	15 (83.3)	117 (75.5)	
> 5	35 (25.5)	3 (16.7)	38 (24.5)	
**Educational level**				0.690
< SHS/Tech/Voc.	117 (85.4)	16 (88.9)	133 (85.8)	
≥ SHS/Tech/Voc.	20 (14.6)	2 (11.1)	22 (14.2)	
**Frequency of carting**				0.155
Once daily	123 (89.8)	18 (100.0)	141 (91.0)	
> once daily	14 (10.2)	0 (0.0)	14 (9.0)	
**Sore throat**				0.951
No	98 (71.5)	13 (72.2)	111 (71.6)	
Yes	39 (28.5)	5 (27.8)	44 (28.4)	
**Coughing**				0.693
No	77 (56.2)	11 (61.1)	88 (56.8)	
Yes	60 (43.8)	7 (38.9)	67 (43.2)	
**Difficulty in breathing**				0.246
No	116 (84.7)	17 (94.4)	133 (85.8)	
Yes	21 (15.3)	1 (5.6)	22 (14.2)	
**Persistent cough**				0.552
No	133 (97.1)	17 (94.4)	150 (96.8)	
Yes	4 (2.9)	1 (5.6)	5 (3.2)	
**Chest pain**				0.712
No	126 (92.0)	17 (94.4)	143 (92.3)	
Yes	11 (8.0)	1 (5.6)	12 (7.7)	
**Chronic mucus**				0.584
No	124 (90.5)	17 (94.4)	141 (91.0)	
Yes	13 (9.5)	1 (5.6)	14 (9.0)	
**Coughing up blood**				**0.006** **
No	137 (100.0)	17 (94.4)	154 (99.4)	
Yes	0 (0.0)	1 (5.6)	1 (0.6)	

**, expresses significant difference

### Detection and distribution of respiratory pathogens

The overall prevalence of respiratory pathogens was 20 (12.9%). Out of the 20 positive samples, SARS-CoV-2 was detected in 10 (6.5%) of the participants. Similarly, *Streptococcus pneumoniae* was detected in the remaining 10 (6.5%) participants. A total of 2 (1.3%) of the study participants had SARS-CoV-2 and *Streptococcus pneumoniae* detected in them. None of the participants tested positive for H_3_N_2_ (**Table 2**).

Participants aged < 24 years had a higher number of respiratory pathogens (11; 61.1%) compared to those who were ≥ 24 years (7; 38.9%). However, this difference was not statistically significant (p = 0.132). The highest number of pathogens was detected in symptomatic patients. Among symptomatic individuals, cough, sore throat, difficulty in breathing, chronic mucus, chest pain and persistent cough were not significantly associated with the detection of respiratory pathogens (p > 0.05). However, coughing up blood was significantly associated with the detection of respiratory pathogens (p = 0.006).

### The use of personal protective equipment

The use of PPE among motor tricycle operators was fairly high. Of the 155 subjects surveyed, 117 (75.5%) indicated they use gloves, 110 (71.0%) use nose masks and 40 (25.8%) use coveralls. There was a statistically significant relationship between the use of gloves and detecting a respiratory pathogen (p = 0.008). Similarly, the use of multiple (>1) PPE was statistically associated with respiratory pathogen detection (p = 0.008). However, the use of nose masks (p = 0.669) and coverall (p = 0.346) was not significantly associated with the detection of respiratory pathogens (**Table 3**).

**Table 3:** Exposure to pollutants and the use of PPE.

	Negative	Positive	Total	*p-value*
Total	137	18	155	
**Use of Gloves**				**0.008** **
No	29 (21.2)	9 (50.0)	38 (24.5)	
Yes	108 (78.8)	9 (50.0)	117 (75.5)	
**Use of nose mask**				0.669
No	39 (28.5)	6 (33.3)	45 (29.0)	
Yes	98 (71.5)	12 (67.7)	110 (71.0)	
**Coverall**				0.346
No	100 (73.0)	15 (83.3)	115 (74.2)	
Yes	37 (27.0)	3 (16.7)	40 (25.8)	
**Use of at least one PPE**				0.477
No	15 (10.9)	3 (16.7)	18 (11.6)	
Yes	122 (89.1)	15 (83.3)	137 (88.4)	
**Use of multiple (> 1) PPE**				**0.008** **
No	41 (29.9)	11 (61.1)	52 (33.5)	
Yes	96 (70.1)	7(38.9)	103 (66.5)	
**Exposure to Dust**				0.827
No	41 (29.9)	11 (61.1)	52 (33.5)	
Yes	96 (70.1)	7(38.9)	103 (66.5)	
**Exposure to Smoke**				0.461
No	95 (69.3)	14 (77.8)	109 (70.3)	
Yes	42 (30.7)	4 (88.9)	46 (29.7)	
**Exposure to other pollutants**				**0.015** **
No	91 (66.4)	17 (94.4)	108 (69.7)	
Yes	46 (33.6)	1 (5.6)	47 (30.3)	

** expresses significant difference

### Exposure to pollutants

**Table 3** gives details about the statistical association between participants’ exposure to dust, smoke, and other pollutants. There was no statistical relationship between exposure to smoke and the detection of respiratory pathogens (p = 0.461). Similarly, exposure to dust had no significant relationship with respiratory pathogens detection (p = 0.461). However, exposure to pollutants other than smoke and dust was statistically significant against respiratory pathogens (p = 0.026).

### Risk factors associated with respiratory infections

**Table 4** shows the independent risk factors associated with detection of respiratory pathogens when a logistic regression model was fitted with respiratory pathogen detection being the dependent variable. There were lower odds of detecting respiratory pathogens in waste tricycle operators who wear gloves. Similarly, the use of more than one PPE (multiple PPE) also reduced the odds of detection of respiratory pathogens (Adj. OR (95% CI): 0.446 (0.093–2.150). Individuals who were exposed to “other pollutants” significantly had lower odds of having respiratory pathogens detected in them (Adj. OR (95% CI): 0.119 (0.015–0.938).

**Table 4:** Risk factors associated with detection of respiratory pathogens.

Variables	Crude OR (95% CI)	Adjusted OR (95% CI)	*p-value*
Use of gloves: Yes vs No	0.269 (0.098–0.738)	0.513 (0.107–2.473)	0.513
Use of multiple (> 1) PPE: Yes vs No	0.272 (0.098–0.750)	0.446 (0.093–2.150)	0.315
Exposure to other pollutants: Yes vs No	0.116 (0.015–0.902)	0.119 (0.015–0.938)	**0.043** **

** expresses significant difference

## Discussion

A cross-sectional study among solid waste motor tricycle operators was conducted to determine the burden of respiratory pathogens and associated factors. The findings showed that the prevalence of respiratory pathogens among solid waste motor tricycle operators was 12.9%. The study also indicated that the most common self-reported symptoms were cough, sore throat and difficulty in breathing. From the analysis, it was observed that the use of gloves, the use of more than one PPE was associated with protection and exposure to other pollutants was significantly associated with testing positive for respiratory pathogens.

A total of 10 (6.5%) participants tested positive for SARS-CoV-2. This is lower than rates reported by a 2020 study on SARS-CoV-2 prevalence in 12 regions of Ghana (13.2%) [14]. This prevalence is however similar to the 6.7% detection rates reported by the Ghana Health Service [15]. The low occurrence of SARS-CoV-2 recorded in this study is not surprising because of a fairly high adherence rate to using PPE such as nose masks (71%) and gloves (75.5%) among participants. *Streptococcus pneumoniae* was also found in 10 (6.5%) of the study participants in this study. This is much lower than recent rates of *S*. *pneumoniae* among children in Kumasi and different parts of the world [16–20]. The disparity is primarily due to the difference in the age distribution of the study participants. The current study recruited adults while the other studies recruited children who have been found to have a significantly higher pneumococcal carriage compared to adults [21]. None of the participants in this study tested positive for Influenza A virus (H_3_N_2_). This is inconsistent with a study that found 41.9% of H_3_N_2_ cases recorded in a study of Influenza outbreaks among students in Kumasi [22]. The Kumasi study was an outbreak investigation, which could account for the relatively high incidence rates. The low positivity rate observed in this study could be due to the constant exposure to viruses, thus, the development of immunity to those viruses [23]. Earlier studies in Ghana have found that most respiratory pathogens circulate during the wet season from March to November [8, 24, 25]. The seasonality of respiratory pathogens may explain the general low detection of respiratory pathogens (12.9%) in this study since the study participants were recruited in the late dry season in February.

Bacterial co-infections are common with respiratory viral pathogens [26]. In fact, in the previous influenza pandemics, *Streptococcus pneumoniae* was the most isolated bacterial pathogen causing increased mortality in patients (22). This study found a co-infection rate of 1.3%, all between SARS-CoV-2 and *S*. *pneumoniae*. This is similar to rates observed in studies in Spain, the USA and Italy which found co-infection rates of 1.2% [27], 1.2% [28] and 0.8% [29] respectively. The presence of respiratory viral infections may create a favourable condition for colonizing pneumococci [30, 31].

The respiratory symptoms reported by participants include sore throat, cough, difficulty in breathing, persistent cough, chest pain, chronic mucus, and coughing up blood. Several African studies among municipal solid waste workers [32], door-to-door solid waste collectors and street sweepers [33] and landfill waste recyclers [34], waste workers have reported similar symptoms [32–34]. Respiratory symptoms generally indicate respiratory disorders including respiratory infections [35]. However, asymptomatic operators’ risk to testing positive for respiratory pathogens was higher [24]. This phenomenon has been reported in several studies [36, 37]. This could be due to the use of increasingly sensitive techniques in the identification of respiratory viruses, such as PCR. The high sensitivity of this test challenges clinical interpretation because it detects the presence of minute amounts of the target organism that may or may not cause clinical symptoms [38].

The current study found a fairly high adherence to the use of PPE by solid waste tricycle operators whiles working. This is in contrast to a study that assessed the occupational health and safety of solid waste tricycle operators within the Kumasi Metropolis. The study found a low adherence rate to the use of PPE (32.5%) [5]. The disparity could be because this study was conducted during the height of the COVID-19 wave and people adhered to the safety protocols. Discussion with the participants who did not use PPE revealed that using PPEs was time-wasting and inconvenient (according to them slowed down their daily activities). The current study revealed that the use of gloves significantly reduced the odds of testing positive for a respiratory pathogen, indicating some level of protection. This is because gloves protect the hands from aerosols containing respiratory pathogens. This prevents self-inoculation. Nose masks prevent the inhalation of pathogen-containing aerosols and thus protect against respiratory infections [39–41]. This was observed in this study as solid waste tricycle operators who use nose masks were less likely to test positive for respiratory pathogens, but the relationship between the use of nose masks and respiratory infection was not significant. Solid waste motor tricycle operators who use multiple PPE (more than 1 PPE) had lower odds of being infected with a respiratory pathogen. This may be because the components of the multiple PPE have all been shown to confer some level of protection against respiratory infection [32, 42].

Workers in solid waste management may be exposed to bioaerosols and organic dust containing various harmful pathogens, as well as to smoke and dust [43, 44]. The collection and transportation solid waste from homes and commercial areas to landfill sites by participants using tricycles, exposes them to smoke, especially from other vehicles as well as smoke at the landfill sites from burning refuse [45]. However, less than a third of the participants perceived exposure to smoke. This study also revealed that exposure to smoke was not significantly associated with respiratory pathogens positivity, which is inconsistent with studies concerning exposure to smoke and respiratory infection [46, 47]. Exposure to other forms of pollutants (nitrogen dioxide (NO_2_) and carbon monoxide (CO) reduced the odds of testing positive for respiratory pathogens although by conventional knowledge, we expect that exposure increases the risk [48]. This can be attributed to the fact that this study accepted subject responses on pollution exposure at face value, which may be prone to recall bias, thus the extent and duration of exposure was not known, which we were unable to elucidate in this study.

One limitation of the study was our inability to test for more respiratory pathogens. Future studies should consider increasing the panel of respiratory pathogens tested. This study was cross-sectional. hence, the prevalence of self-reported symptoms and respiratory pathogens was studied at a specific point in time. Future studies should consider a longitudinal design where selected participants will be surveyed for symptoms and respiratory pathogens over time. This will give a better understanding of the incidence of respiratory pathogens among waste workers.

## Conclusion

The presence of respiratory symptoms and pathogens among solid waste motor tricycle operators in the Kumasi metropolis has been demonstrated. It has also been established that the usage of gloves and multiple PPEs protect against respiratory pathogens. The Kumasi Metropolitan Assembly through her agents at the landfill site should educate and enforce the use of PPEs by the tricycle operators for their own safety.

## Supporting information

S1 File (DOCX)

## References

[1] Jack JKA, Amoah EK, Hope E, Okyere F. Should Ghana Legalize the Commercial Use of Motor Bikes and Tricycles as Means of Public Transport? A Case Study of Five Selected Regions in Ghana. Journal of Economics and Business. 2021;4(1).

[2] Dinye R. The significance and issues of motorcycle transport in the Urban areas in northern Ghana. Scientific Journal of review. 2013;2(10):256–72.

[3] Sarfo-Mensah P, Obeng-Okrah K, Arhin AA, Amaning TK, Oblitei RT. Solid waste management in urban communities in Ghana: A case study of the Kumasi metropolis. African Journal of Environmental Science and Technology. 2019;13(9):342–53.

[4] Issahaku I, Nyame FK, Brimah AK. Waste management strategies in an urban setting example from the Tamale Metropolis, Ghana. Journal of Waste Management. 2014;2014.

[5] Asibey MO, Amponsah O, Yeboah V. Solid waste management in informal urban neighbourhoods. Occupational safety and health practices among tricycle operators in Kumasi, Ghana. International journal of environmental health research. 2019;29(6):702–17. 10.1080/09603123.2019.1569211 30714824

[6] Garrido MV, Bittner C, Harth V, Preisser AM. Health status and health-related quality of life of municipal waste collection workers–a cross-sectional survey. Journal of Occupational medicine and toxicology. 2015;10(1):1–7. 26155300 10.1186/s12995-015-0065-6 PMC4493964

[7] Alam P, Ahmade K. Impact of solid waste on health and the environment. International Journal of Sustainable Development and Green Economics (IJSDGE). 2013;2(1):165–8.

[8] Annan A, Ebach F, Corman V, Krumkamp R, Adu-Sarkodie Y, Eis-Hübinger A, et al. Similar virus spectra and seasonality in paediatric patients with acute respiratory disease, Ghana and Germany. Clinical Microbiology and Infection. 2016;22(4):340–6. doi: 10.1016/j.cmi.2015.11.002 26585774 PMC7172147

[9] Albuquerque RC, Moreno ACR, Dos Santos SR, Ragazzi SLB, Martinez MB. Multiplex-PCR for diagnosis of bacterial meningitis. Brazilian Journal of Microbiology. 2019;50(2):435–43. doi: 10.1007/s42770-019-00055-9 30796713 PMC6863191

[10] van Elden LJ, Nijhuis M, Schipper P, Schuurman R, van Loon AM. Simultaneous detection of influenza viruses A and B using real-time quantitative PCR. Journal of clinical microbiology. 2001;39(1):196–200. Epub 2001/01/04. doi: 10.1128/JCM.39.1.196-200.2001 ; PubMed Central PMCID: PMC87701. 11136770 PMC87701

[11] Corman VM, Landt O, Kaiser M, Molenkamp R, Meijer A, Chu DK, et al. Detection of 2019 novel coronavirus (2019-nCoV) by real-time RT-PCR. Eurosurveillance. 2020;25(3):2000045. doi: 10.2807/1560-7917.ES.2020.25.3.2000045 31992387 PMC6988269

[12] CDC. Research use only 2019-novel coronavirus (2019-nCoV) real-time RT-PCR primer and probe information 2020.

[13] Chen Y, Cui D, Zheng S, Yang S, Tong J, Yang D, et al. Simultaneous detection of influenza A, influenza B, and respiratory syncytial viruses and subtyping of influenza A H3N2 virus and H1N1 (2009) virus by multiplex real-time PCR. Journal of clinical microbiology. 2011;49(4):1653–6. doi: 10.1128/JCM.02184-10 21270233 PMC3122825

[14] Owusu M, Sylverken AA, Ankrah ST, El-Duah P, Ayisi-Boateng NK, Yeboah R, et al. Epidemiological profile of SARS-CoV-2 among selected regions in Ghana: A cross-sectional retrospective study. PLoS One. 2020;15(12):e0243711. doi: 10.1371/journal.pone.0243711 33301533 PMC7728229

[15] GHS. Situation Update, Covid-19 Outbreak In Ghana As At 12 December 2021 2021 [cited 19 December, 2021 19 December, 2021]. Available from: https://www.ghs.gov.gh/covid19/.

[16] Narwortey DK, Owusu-Ofori A, Slotved H-C, Donkor ES, Ansah PO, Welaga P, et al. Nasopharyngeal carriage of Streptococcus pneumoniae among healthy children in Kassena-Nankana districts of Northern Ghana. BMC Infectious Diseases. 2021;21(1):1–10. 34233627 10.1186/s12879-021-06302-5 PMC8265090

[17] Haile AA, Gidebo DD, Ali MM. Colonization rate of Streptococcus pneumoniae, its associated factors and antimicrobial susceptibility pattern among children attending kindergarten school in Hawassa, southern Ethiopia. BMC Research Notes. 2019;12(1):1–7. 31208447 10.1186/s13104-019-4376-z PMC6580519

[18] Salsabila K, Paramaiswari WT, Amalia H, Ruyani A, Tafroji W, Winarti Y, et al. Nasopharyngeal carriage rate, serotype distribution, and antimicrobial susceptibility profile of Streptococcus pneumoniae isolated from children under five years old in Kotabaru, South Kalimantan, Indonesia. Journal of Microbiology, Immunology and Infection. 2022;55(3):482–8. doi: 10.1016/j.jmii.2021.06.006 34294592

[19] Adetifa IM, Adamu AL, Karani A, Waithaka M, Odeyemi KA, Okoromah CA, et al. Nasopharyngeal pneumococcal carriage in Nigeria: a two-site, population-based survey. Scientific reports. 2018;8(1):1–9. 29472635 10.1038/s41598-018-21837-5 PMC5823928

[20] Denno DM, Frimpong E, Gregory M, Steel RW. Nasopharyngeal carriage and susceptibility patterns of Streptococcu Pneumoniae in Kumasi, Ghana. West African Journal of Medicine. 2002;21(3):233–6. 12744576

[21] Irfan O, Li J, Tang K, Wang Z, Bhutta ZA. Risk of infection and transmission of SARS-CoV-2 among children and adolescents in households, communities and educational settings: A systematic review and meta-analysis. Journal of Global Health. 2021;11. doi: 10.7189/jogh.11.05013 34326997 PMC8285769

[22] Sylverken AA, Owusu M, Yeboah R, El-Duah P, Gorman R, Bonney JK, et al. Influenza outbreak among students in Ghana: a report from three time points. 2020.

[23] Musher DM. How contagious are common respiratory tract infections? New England Journal of Medicine. 2003;348(13):1256–66. doi: 10.1056/NEJMra021771 12660390

[24] Kwofie TB, Anane YA, Nkrumah B, Annan A, Nguah SB, Owusu M. Respiratory viruses in children hospitalized for acute lower respiratory tract infection in Ghana. Virology journal. 2012;9(1):1–8. doi: 10.1186/1743-422X-9-78 22490115 PMC3364910

[25] Obodai E, Odoom JK, Adiku T, Goka B, Wolff T, Biere B, et al. The significance of human respiratory syncytial virus (HRSV) in children from Ghana with acute lower respiratory tract infection: a molecular epidemiological analysis, 2006 and 2013–2014. PLoS One. 2018;13(9):e0203788. doi: 10.1371/journal.pone.0203788 30199549 PMC6130863

[26] Pal C, Przydzial P, Chika-Nwosuh O, Shah S, Patel P, Madan N. Streptococcus pneumoniae coinfection in COVID-19: a series of three cases. Case Reports in Pulmonology. 2020;2020. doi: 10.1155/2020/8849068 33343959 PMC7729390

[27] Garcia-Vidal C, Sanjuan G, Moreno-García E, Puerta-Alcalde P, Garcia-Pouton N, Chumbita M, et al. Incidence of co-infections and superinfections in hospitalized patients with COVID-19: a retrospective cohort study. Clinical Microbiology and Infection. 2021;27(1):83–8. doi: 10.1016/j.cmi.2020.07.041 32745596 PMC7836762

[28] Lehmann CJ, Pho MT, Pitrak D, Ridgway JP, Pettit NN. Community-acquired coinfection in coronavirus disease 2019: a retrospective observational experience. Clinical Infectious Diseases. 2021;72(8):1450–2. doi: 10.1093/cid/ciaa902 32604413 PMC7337635

[29] Bordi L, Nicastri E, Scorzolini L, Di Caro A, Capobianchi MR, Castilletti C, et al. Differential diagnosis of illness in patients under investigation for the novel coronavirus (SARS-CoV-2), Italy, February 2020. Eurosurveillance. 2020;25(8):2000170. doi: 10.2807/1560-7917.ES.2020.25.8.2000170 32127123 PMC7055037

[30] Howard LM. Is There an Association Between Severe Acute Respiratory Syndrome Coronavirus 2 (SARS-CoV-2) and Streptococcus pneumoniae?: Oxford University Press US; 2021. 10.1093/cid/ciaa1812 PMC7799239 33274382

[31] Bosch AA, Biesbroek G, Trzcinski K, Sanders EA, Bogaert D. Viral and bacterial interactions in the upper respiratory tract. PLoS pathogens. 2013;9(1):e1003057. doi: 10.1371/journal.ppat.1003057 23326226 PMC3542149

[32] Emiru Z, Gezu M, Chichiabellu TY, Dessalegn L, Anjulo AA. Assessment of respiratory symptoms and associated factors among solid waste collectors in Yeka Sub City, Addis Ababa, Ethiopia. Journal of Public Health and Epidemiology. 2017;9(6):189–97.

[33] Eneyew B, Sisay T, Gizeyatu A, Lingerew M, Keleb A, Malede A, et al. Prevalence and associated factors of acute respiratory infection among street sweepers and door-to-door waste collectors in Dessie City, Ethiopia: A comparative cross-sectional study. PloS one. 2021;16(5):e0251621. doi: 10.1371/journal.pone.0251621 33989364 PMC8121341

[34] Tlotleng N, Kootbodien T, Wilson K, Made F, Mathee A, Ntlebi V, et al. Prevalence of respiratory health symptoms among landfill waste recyclers in the city of Johannesburg, South Africa. International journal of environmental research and public health. 2019;16(21):4277. doi: 10.3390/ijerph16214277 31689929 PMC6862197

[35] Thompson M, Vodicka TA, Blair PS, Buckley DI, Heneghan C, Hay AD. Duration of symptoms of respiratory tract infections in children: systematic review. Bmj. 2013;347:f7027. doi: 10.1136/bmj.f7027 24335668 PMC3898587

[36] Herberhold S, Eis-Hübinger A-M, Panning M. Frequent detection of respiratory viruses by real-time PCR in adenoid samples from asymptomatic children. Journal of clinical microbiology. 2009;47(8):2682–3. 19494063 10.1128/JCM.00899-09 PMC2725659

[37] Graat JM, Schouten EG, Heijnen M-LA, Kok FJ, Pallast EG, de Greeff SC, et al. A prospective, community-based study on virologic assessment among elderly people with and without symptoms of acute respiratory infection. Journal of clinical epidemiology. 2003;56(12):1218–23. doi: 10.1016/s0895-4356(03)00171-9 14680673 PMC7119012

[38] Jansen RR, Wieringa J, Koekkoek SM, Visser CE, Pajkrt D, Molenkamp R, et al. Frequent detection of respiratory viruses without symptoms: toward defining clinically relevant cutoff values. Journal of clinical microbiology. 2011;49(7):2631–6. doi: 10.1128/JCM.02094-10 21543571 PMC3147826

[39] Abboah-Offei M, Salifu Y, Adewale B, Bayuo J, Ofosu-Poku R, Opare-Lokko EBA. A rapid review of the use of face mask in preventing the spread of COVID-19. International journal of nursing studies advances. 2021;3:100013. doi: 10.1016/j.ijnsa.2020.100013 33313575 PMC7718106

[40] Greenhalgh T, Schmid MB, Czypionka T, Bassler D, Gruer L. Face masks for the public during the covid-19 crisis. Bmj. 2020;369. doi: 10.1136/bmj.m1435 32273267

[41] Teleman MD, Boudville I, Heng B, Zhu D, Leo Y. Factors associated with transmission of severe acute respiratory syndrome among health-care workers in Singapore. Epidemiology & Infection. 2004;132(5):797–803. doi: 10.1017/s0950268804002766 15473141 PMC2870165

[42] Chughtai AA, Khan W. Use of personal protective equipment to protect against respiratory infections in Pakistan: A systematic review. Journal of infection and public health. 2020;13(3):385–90. doi: 10.1016/j.jiph.2020.02.032 32146139 PMC7102706

[43] Carducci A, Federigi I, Verani M. Virus occupational exposure in solid waste processing facilities. The Annals of occupational hygiene. 2013;57(9):1115–27. Epub 2013/08/07. doi: 10.1093/annhyg/met043 . 23917836

[44] Park D-U, Ryu S-H, Kim S-B, Yoon C-S. An assessment of dust, endotoxin, and microorganism exposure during waste collection and sorting. Journal of the Air & Waste Management Association. 2011;61(4):461–8. 10.3155/1047-3289.61.4.461 21516941

[45] Akormedi M, Asampong E, Fobil JN. Working conditions and environmental exposures among electronic waste workers in Ghana. International Journal of Occupational and Environmental Health. 2013;19(4):278–86. doi: 10.1179/2049396713Y.0000000034 24588034

[46] Jones LL, Hashim A, McKeever T, Cook DG, Britton J, Leonardi-Bee J. Parental and household smoking and the increased risk of bronchitis, bronchiolitis and other lower respiratory infections in infancy: systematic review and meta-analysis. Respiratory research. 2011;12(1):1–11. 21219618 10.1186/1465-9921-12-5 PMC3022703

[47] Tazinya AA, Halle-Ekane GE, Mbuagbaw LT, Abanda M, Atashili J, Obama MT. Risk factors for acute respiratory infections in children under five years attending the Bamenda Regional Hospital in Cameroon. BMC pulmonary medicine. 2018;18(1):1–8. 29338717 10.1186/s12890-018-0579-7 PMC5771025

[48] Brugha R, Grigg J. Urban air pollution and respiratory infections. Paediatric respiratory reviews. 2014;15(2):194–9. doi: 10.1016/j.prrv.2014.03.001 24704510

