# Supplementary material for: Solid waste motor tricycle operators in Kumasi, Ghana, harbour respiratory pathogens; a public health threat
Source: PLoS One. 2023 Apr 24;18(4):e0284985. doi: 10.1371/journal.pone.0284985 (PMC10124853; doi:10.1371/journal.pone.0284985)
Supplement: S1 File — (DOCX) [file pone.0284985.s001.docx]

Participant ID………………

**QUESTIONNAIRE**

Kindly answer the following questions in this questionnaire to enable us conduct this study

- **SECTION A: Socio-demographic Characteristics**

1. Age ……………………………
2. Gender

a) male [ ] b) female [ ]

1. Marital Status

a) Single [ ] b) Married [ ] c) Divorced [ ] d) Others [ ]

if others, please specify ……………………………………………

1. Educational level

a) None [ ] b) Primary [ ] c) MSLC/JSS [ ] d) SSS/Voc./Tech [ ] e) Diploma [ ] f) Bachelor’s degree [ ] g) Master’s degree [ ]

1. How long (years) have you worked as a motor tricycle operator?

a) 5 or less [ ] b) 6-10 [ ] c) 11-15 [ ] d) 16-20 [ ] e) above 20 [ ]

**6.** What type of house do you live in?

a) Detached house [ ] b) Semi-detached houses [ ] c) Apartment houses [ ]

d) compound houses [ ] e) Others [ ] if others, please specify…………………….

1. How many people are sleeping in a room in your house?
2. One [ ] b) Two [ ] c) Three [ ] d) More than three [ ]
3. How many people are in living in your household? [**indices of crowding**]
4. One [ ] b) Two [ ] c) Three [ ] d) Four [ ] e) Five [ ]

f) Others [ ] if others, please specify ……………………………………………

- **SECTION B: Frequency of carting refuse**

1. How many times in a **day** do you cart refuse from collection to dumping points?

a) Once [ ] b) Twice [ ] c) Thrice [ ] d) More than thrice [ ]

1. Which part(s) of Kumasi (KMA) do you cart the refuse to and from?

…………………………………………………………………………..

1. Which time of the day do you normally cart the refuse?

- Starting time

a) Before 6:00 am [ ] b) Between 6:00 am to 11:59am [ ] c) 12:00 pm to 4:00 pm [ ]

d) Between 4:00 pm to 6:00 pm [ ] e) After 6:00 pm [ ]

- Closing time

a) Before 6:00 am [ ] b) Between 6:00 am to 11:59am [ ] c) 12:00 pm to 4:00 pm [ ]

d) Between 4:00 pm to 6:00 pm [ ] e) After 6:00 pm [ ]

- **SECTION C: Safety compliance**

1. Which personal protective equipments (PPEs) do you have access to?

| **Access/possession** | | |  |  |  |
| --- | --- | --- | --- | --- | --- |
| **PPEs** | **Yes** | **No** | | **How many days in a week do you wear them?** | |
| Gloves |  |  | |  | |
| Nose mask |  |  | |  | |
| Face shield |  |  | |  | |
| Coverall |  |  | |  | |
| Boots |  |  | |  | |
| Helmet |  |  |  | |  |
| Googles |  |  |  | |  |
| Others |  |  |  | |  |

1. What are some of the reasons why you don’t wear them?

a) Time wasting [ ] b) Does not fit [ ] c) Don’t know how to wear [ ]

d) I cannot hear [ ] e) I cannot see [ ] f) I cannot breath [ ]

g) if others, please specify ………………………………………………………….

- **SECTION D: Respiratory disorders**

1. Which of the following have you experienced recently (past 2 months)? (Can be more than one)
2. Sore throat [ ]
3. Coughing [ ]
4. Difficulty in breathing [ ]
5. Persistent cough [ ]
6. Lingering Chest pain [ ]
7. Chronic mucus [ ]
8. Coughing up blood [ ]
9. Have you ever been diagnosed of Tuberculosis before?
10. Yes [ ] b) No [ ]
11. Have you ever been hospitalized as a result of any respiratory disorder before?
12. Yes [ ] b) No [ ]
13. Has a close relative (family) been exposed to any respiratory infection recently (past month)?
14. Yes [ ] b) No [ ]
15. Have you been diagnosed with HIV/AIDS before?
16. Yes [ ] b) No [ ]

- **SECTION E: Behavioural factors**

1. Do you take alcoholic beverages?
2. Yes [ ] b) No [ ]
3. If yes, how often do you take it??
4. Daily [ ] b) Weekly [ ] c) Monthly [ ] d) Occasionally [ ]
5. Do you smoke cigarettes/tobacco/ other ‘smokables’?

a) Yes, currently [ ] b)No, lives with a smoker [ ] c)No, Used to smoke [ ]

d) Never smoked [ ]

22. If you currently smoke, how often do you do it?

1. Daily [ ] b) Weekly [ ] c) Monthly [ ] d) Occasionally [ ]

23. If you live with a smoker, are you exposed to it?

a) Yes [ ] b) No [ ]

1. How often do you exercise?
2. Daily [ ] b) Weekly [ ] c) Monthly [ ] d) Occasionally [ ] e) Never [ ]
3. Do you have any allergies?
4. Yes [ ] b) No [ ]
5. Are you frequently exposed to the following?
6. Dust [ ] b) Smoke [ ] c) Other pollutants [ ]
